# Iron intake and iron status of Swedish adolescents with diets of varying climate impact

**DOI:** 10.1007/s00394-024-03572-y

**Published:** 2025-02-15

**Authors:** Elinor Hallström, Josefin Edwall Löfvenborg, Lotta Moreaus, Agneta Sjöberg, Anna Winkvist, Anna Karin Lindroos

**Affiliations:** 1 Nutrition, Sustainability and Health Promotion Group, National Food Institute, Technical University of Denmark, Kgs Lyngby, Denmark; 2 Department of Food and Agriculture, Research Institutes of Sweden (RISE), Lund, Sweden; 3 Division for Risk and Benefit Assessment, Swedish Food Agency, Uppsala, Sweden; 4 Department of Food and Nutrition and Sport Science, University of Gothenburg, Gothenburg, Sweden; 5 Department of Internal Medicine and Clinical Nutrition, the Sahlgrenska Academy, University of Gothenburg, Gothenburg, Sweden

**Keywords:** Iron deficiency, Nutrition, Environmental impact, Sustainable diets, Life cycle assessment, Adolescents

## Abstract

**Purpose:**

The risk of inadequate micronutrient intake is a concern of low-climate impact diets. This study analyzes the prevalence of iron deficiency (ID) among adolescents with varying dietary climate impact, with special reference to different types and dietary sources of iron.

**Methods:**

Data on dietary intake (*n* = 3099) and plasma ferritin (*n* = 1030) were from Riksmaten Adolescents 2016–2017 survey of Swedish girls and boys aged 11–18 years. Dietary climate impact was estimated with life cycle assessment data. Linear and logistic regression models assessed associations between dietary climate impact, intakes of iron and food groups, and ID.

**Results:**

Higher total iron and heme, but not non-heme, iron intake, was linearly associated with higher dietary climate impact. Compared to girls, boys had higher climate impact and low prevalence of ID. Girls in the highest climate impact quartile had 56% lower odds of ID (OR 0.44, 95% CI 0.24–0.81) compared to the lowest quartile, whereas no association was found in boys. Lower intake of red meat and heme iron was associated with ID in girls, while higher intake of dairy was associated with ID in boys. Menstruating girls and adolescents born outside of Sweden were identified risk groups for ID.

**Conclusions:**

Girls with a more climate-friendly diet and lower intake of red meat/heme iron may be at higher risk of ID compared to girls with higher dietary climate impact. These results highlight the importance of considering risk groups of ID, such as menstruating girls, in the transition to more plant-based diets with lower climate impact.

**Supplementary Information:**

The online version contains supplementary material available at 10.1007/s00394-024-03572-y.

## Introduction

In affluent countries like Sweden current dietary patterns are in many ways linked to health and environmental challenges [1–3]. Dietary change is proposed to have major potential for contributing to improved health and reduced environmental impact [4]. However, trade-offs between health and environmental aspects may occur [5]. An increased proportion of plant-based foods is suggested as a key strategy for sustainable diets [6]. However, concerns have been raised about the risk of lower intake and uptake of micronutrients in climate-adapted diets with lowered amounts of meat and other animal-based foods [7]. Iron is of special concern due to its generally low bioavailability in plant-based foods and the high prevalence of iron deficiency (ID) in both low- and high-income countries, especially among children, and fertile and pregnant women [8, 9].

Insufficient intake of iron and/or low bioavailability can reduce iron stores and ultimately lead to ID when stores are inadequate to meet physiological requirements. Iron deficiency can further lead to reduced haemoglobin levels and is the main cause of anemia globally [8]. Serum/plasma ferritin is considered the best biomarker of iron status, indicating the size of iron stores [10]. The bioavailability of iron is affected by several factors, including the type of iron (i.e., heme and nonheme), the presence of iron uptake inhibitors (e.g., phytic acid, polyphenols, calcium) and enhancers (e.g., ascorbic acid, animal tissues) in the diet, and the current physiological iron status [11, 12]. Although these factors may substantially affect the iron status of individuals, the nutrient adequacy of diets varying in climate impact has mainly been assessed based on the nutrient intake, without considering nutrient status or the mechanisms affecting absorption [13], which may have implications for the results and conclusions.

Iron requirements are elevated during adolescence [14] to cover needs for growth and, in girls, to cover iron losses due to menstruation [8]. In Sweden, 10–26% of adolescent girls (aged 11–18 years) suffer from ID [15]. A positive association between energy-standardized intake of total iron and dietary climate impact was recently shown among Swedish adolescents [16]. Iron is the nutrient most studied in relation to dietary changes aimed at reducing environmental impact; however, so far, iron status has not been assessed in relation to dietary climate impact, according to a recent review [13]. Moreover, associations between dietary choices, iron intake, and iron status, considering differences between types of iron and population groups, have not been thoroughly assessed. Consequently, the health effects of different iron intakes in risk groups and practical guidance of how to minimize the risk of ID in populations shifting to plant-based diets are highlighted as main data gaps in the latest Nordic Nutrition Recommendations [8], which call for more research. To address these knowledge gaps, the overall aim of this article is to describe the prevalence of ID among Swedish adolescents with varying dietary climate impact, with special reference to different types and dietary sources of iron.

## Method

### Study population

Data were obtained from the Swedish dietary survey Riksmaten Adolescents 2016–2017, a cross-sectional, school-based, nationally representative survey conducted by the Swedish Food Agency between September 2016 and May 2017. The survey included adolescents in school grades 5 (11–12 y), 8 (14–15 y) and 11 (17–18 y). A total of 619 schools were randomly selected from the Swedish school registry by Statistics Sweden to represent pupils in all three school grades across Sweden. The sampling frame consisted of all schools in Sweden with pupils in the specified age groups, excluding schools with fewer than 10 pupils in a school grade and high schools with only language introduction. The selection of schools was based on type of municipality, geographical location, and whether the school was public or independent. All pupils in the selected classes were potential participants. Exclusion criteria included participating in language introduction, special education, and adult education (*n* = 36). In total, 5145 adolescents from 131 schools that agreed to participate were invited, of which 3099 participants provided complete information on diet (60%). In the subgroup also invited to provide blood samples (*n* = 2378), 1105 (46%) participated and provided complete dietary information. The study groups were overall representative of the underlying population regarding socioeconomic background, geographical area, type of municipality, and school organization (public or independent) [17]. A detailed description of the study design, methods, and participation has been provided elsewhere [17]. Ethical approval was obtained from the Regional Ethical Review Board in Uppsala (No. 2015/190).

The main analytical sample comprises those participants in the survey who provided complete dietary information and blood samples. Further exclusions were made due to the unavailability of blood tubes for analysis (*n* = 28) and chronic genetic diseases causing elevated ferritin concentrations (*n* = 1). Participants with C-reactive protein (CRP) ≥ 5 mg/L (*n* = 46) were also excluded from the analyses, as high CRP concentrations indicate an ongoing infection, which can lead to high ferritin values, even though iron stores may be low, and thus, may result in falsely high plasma ferritin values [18]. This left a final sample of 1030 participants, hereafter referred to as the iron status subsample. For the analysis of the contribution of dietary iron from food groups, the full survey sample of 3099 participants was used.

### Dietary data

Food intake was estimated using the web-based dietary assessment method RiksmatenFlex, a self-administered 24-hour recall method assessing intake during two independent days. The method has been validated against independent biomarkers in adolescents, and the results were satisfactory for the intake of energy, wholegrains, fruits and vegetables [19]. Intake was reported from a list of 778 foods and beverages linked to the Swedish Food Agency ‘s Food composition database (version Riksmaten Adolescents 2016–2017), allowing for direct calculation of energy and nutrient intake per day. Portion sizes were estimated using a picture portion guide embedded in the web-based dietary survey, which illustrated different reference sizes for food categories to help participants estimate the amounts consumed. The recall days were checked for completeness according to pre-specified criteria [17].

The heme iron content of foods was estimated, as the Swedish food composition database only provides information on the total iron content. The estimation was based on the total iron content from the meat/fish part of a food and the proportion of heme iron within this total iron. Based on Balder et al. [20], the following heme iron proportions were used for cooked meat/fish products: beef 65%, pork 35%, fish/seafood 26% and poultry 26%. Additionally, 24% was used for liver, 59% for mutton [21], 76% for blood-based foods [22], 57% for minced meat (1/3 pork, 2/3 beef), and 52% for sausages (mean of beef and pork). No data were available for game, so the heme iron proportion of beef (65%) was used. The heme iron content was compiled per 100 g of food, and the intake per day was calculated for each participant by multiplying the reported intake of each food by the heme iron content. Estimated non-heme iron intake per day was calculated as the difference between daily total iron intake and estimated heme iron intake.

Associations between iron status and food intake were assessed for food groups that contributed most to total dietary iron intake in the population. In addition, the intake of dairy products was assessed due to their high contribution of calcium, which may limit iron absorption [23]. Sweet foods and sugar-sweetened beverages were assessed, as high consumption levels of these food groups have been found among Swedish adolescents, particularly among girls in the older age groups, who also have the lowest intake of red meat and the highest iron requirements [16]. Based on this, we wanted to assess whether sweet foods and sugar-sweetened beverages might be dietary risk factors for ID by replacing foods in the diet with higher nutritional value. For these assessments, food intake was grouped into the following food groups: *Red meat*– single foods of red and processed meats plus the meat part of composite dishes; *Fruit and vegetables–* single foods of fruits, berries, vegetables plus the fruit/berry/vegetable part of composite dishes; *Dairy products*– single foods of milk, yoghurt, cheese; *Cereal products–* single foods of bread, breakfast cereals, grains, pasta, rice; *Sweet foods -* candy, chocolate, sweet bread, cakes, cookies/biscuits, desserts, ice cream; *Sugar-sweetened beverages*– soft drinks, squash, and energy drinks.

For the contribution of iron from food groups, the categorization was done at the food level, as nutrient content was only available at this level [19]. Composite foods consisting of multiple ingredients were categorized based on their main food ingredient by weight, and food group intake was aggregated into 34 food groups and 10 main food categories (see Online Resource Table S3).

### Assessment of iron status

Ferritin and CRP in plasma were analyzed using an Abbott Architect ci8200 system (Abbott Laboratories, Abbott Park, IL, USA) at the Department of Clinical Chemistry and Pharmacology, University of Uppsala, and the Academic Hospital, Uppsala, Sweden. The laboratory is certified according to SS-EN ISO/IEC 15,189. The analytical uncertainty (coefficient of variation) for the methods was as follows: ferritin 5% and CRP (turbidimetry) 4–5%. Ferritin < 15 µg/l was used as the cut-off for ID [24].

### Assessment of dietary climate impact

Dietary climate impact was assessed by linking food intake to climate impact data in the Food and Climate Impact Database provided by the Research Institute of Sweden [25]. The database is updated annually and contains climate data for 800 foods, primarily based on published life cycle assessments (LCAs), reported as greenhouse gas emissions per amount of food produced. To account for differences in global warming potential between greenhouse gases, climate impact is reported as carbon dioxide equivalents (CO_2_e), calculated over a 100-year time frame. The system boundaries include greenhouse gas emissions from primary production to industry gate, including emissions from primary production, processing, transportation to the industry gate and transportation to Sweden for imported foods, but excluding emissions for packaging. Differences in climate impact of food items from different production systems and origins were accounted for by using weighted averages of climate impact representing the average Swedish consumption. The weighted averages were calculated by aggregating climate data based on the percentage contribution obtained from national consumption statistics. For an accurate link with estimated intake, the climate impact data was adjusted to report the impact per edible amount of food (e.g., meat without bones, banana without peel) and corrected for weight changes associated with cooking (e.g., hydration of legumes and rice, dehydration of meat and fish). Values for edible shares of food items and weight changes in cooking were based on data from the Swedish Food Agency Food composition database. The climate impact of composite dishes was estimated based on recipes for the included ingredients.

### Other variables

Information on age, sex and school location was collected from class lists. Information on school location was used to classify participants as living in an urban or a rural setting based on the classification of municipalities, as described in more detail by Moraeus et al. [17]. Information on birth country, household education, dietary preferences, and use of supplements was collected via online questionnaires. Participants were classified as being born in Sweden or not based on reported birth country. Household education referred to the highest level of education attained by either parent. The five response levels were dichotomized into ≤ 12 years and > 12 years. The dietary preference response was categorized as either eating meat or omitting meat from the diet. Iron supplement use was defined as reporting the use of an iron supplement, either alone or as part of a multimineral supplement. For the latter, the participant was considered a user only if the brand/name of the multimineral was provided for verification of its iron content.

Weight was measured in light clothing to the nearest 0.1 kg using SECA 862 or 899 digital weighing scales. Height was measured to the nearest 0.1 cm using SECA 213 portable stadiometers. BMI was calculated (kg/m^2^), and weight status was determined using the International Obesity Task Force reference [26].

### Statistical assessments

All analyses were performed using Stata Statistical Software Release 17 (StataCorp, 2021, College Station, TX: StataCorp LLC). Since the reported diet was based on only 2 days, dietary intake and climate impact were transformed from current to long-term intake using the statistical method Multiple Source Method (MSM) [27, 28]. The transformation was stratified by school grade and sex. All participants were assumed to be consumers, except for meat, where participants reporting the omission of meat were treated as non-consumers. The statistical significance level was set at *p* < 0.05, and all analyses were stratified by sex. Baseline characteristics were presented as means with standard deviations (SD) for continuous variables, and proportions were expressed as percentages and numbers of individuals (n) for categorical variables. Characteristics were compared between participants in the subsample providing blood samples and individuals not in the subsample based on the p-value obtained from Student’s t-test for means and χ^2^-test for proportions. Estimated energy-standardized dietary climate impact, expressed in kg CO_2_e per 10 megajoule (MJ), was presented as means (SD), and the association with iron intake and ID, respectively, was investigated in relation to sex-specific quartiles. Means with SD and medians with the 25th and 75th percentiles (p25 and p75) of plasma ferritin concentration and iron intake (in mg/day and mg/10 MJ) were calculated for all participants and per school grade. The latter was also calculated for quartiles of energy-standardized CO_2_e. Differences between group means were assessed using one-way ANOVA.

Unadjusted logistic regression models were used to estimate odds ratios (OR) with 95% confidence intervals (CI) for the association between various background characteristics and ID. ORs with 95% CIs were also estimated for ID in relation to sex-specific quartiles of energy-standardized CO_2_e, intakes of iron, and different food groups, respectively. Two models were specified; one unadjusted model and adjusted model 1, which accounted for menstruation (for girls; yes/no), school grade (grade 5, 8, or 11), and country of birth (Sweden: yes/no). The covariates included were those non-dietary background variables associated with ID in the univariable model described above. For the regression on food groups, an additional adjusted model 2 was specified, which included mutual adjustment for the different food groups (as continuous intake in g/day). In the adjusted models, school was included as a cluster variable to account for intragroup correlations. Linear regression analysis was used to explore the association between energy-standardized CO_2_e and energy-standardized iron intake (mg/10 MJ), with results presented as beta coefficients with 95% CIs and p-values. As a post-hoc sensitivity analysis, restricted to menstruating girls, the association between quartiles of energy-standardized dietary climate impact and ID was explored. In another post-hoc sensitivity analysis, the use of iron supplements was included as a covariate in the logistic regression analysis estimating the OR for ID in relation to dietary climate impact, total iron intake, and red meat intake, respectively.

## Results

### Characteristics of study sample

Background characteristics did not differ between participants included and those not included in the iron status subsample, except for a lower urban residence (*p* = 0.002) and higher energy intake (*p* = 0.007) in boys, as well as slightly higher age (*p* = 0.035) in girls, in the subsample (Table 1).

**Table 1:** Characteristics of participants in Riksmaten Adolescents 2016–17, both in full and subdivided into the iron status subsample and those without available iron status data (i.e., not in subsample)

	Full Riksmaten sample		Subsample		Not in subsample	
	Girls *n* = 1710	Boys *n* = 1389	Girls *n* = 579	Boys *n* = 451	Girls *n* = 1131	Boys *n* = 938
Age, yrs, mean (SD)	14.6 (2.6)	14.5 (2.6)	14.8 (2.5)	14.4 (2.5)	14.5 (2.6)	14.5 (5.6)
Menarche, % (n) menstruating^a^	68 (1146)	NA	71 (407)	NA	66 (739)	NA
Overweight/obese^b^, % (n)	21 (353)	21 (293)	20 (115)	21 (95)	21 (238)	21 (198)
Household education > 12 yrs, % (n)^c^	62 (1010)	60 (771)	61 (338)	63 (267)	63 (672)	59 (504)
Born outside Sweden, % (n)^d^	10 (166)	12 (171)	10 (60)	12 (56)	9 (106)	12 (115)
Urban residence, % (n)	66 (1125)	71 (984)	63 (366)	65 (295)	67 (759)	73 (689)
Energy intake, MJ, mean (SD)	8.0 (1.8)	10.0 (2.8)	8.1 (1.7)	10.3 (2.6)	8.0 (1.8)	9.8 (2.8)
Protein, E%, mean (SD)	16 (3)	18 (3)	16 (3)	18 (3)	16 (3)	18 (3)
Total fat, E%, mean (SD)	36 (4)	35 (4)	36 (4)	35 (4)	36 (4)	35 (4)
Carbohydrates, E%, mean (SD)	47 (5)	46 (5)	47 (5)	45 (5)	47 (5)	46 (5)

^a^ Data missing for *n* = 16 girls, of which *n* = 9 in the subsample

^b^ Data missing for *n* = 12 girls and *n* = 14 boys, of which none in the subsample

^c^ Data missing for *n* = 85 girls and *n* = 109 boys, of which *n* = 26 girls and *n* = 24 boys in the subsample

^d^ Data missing for *n* = 5 girls and *n* = 9 boys, of which *n* = 2 girls and no boys in the subsample

### Iron intake and status in Swedish adolescents

#### Iron intake

Table 2 presents the intake of total iron, non-heme iron, and heme iron for girls and boys in different age groups of the iron status subsample. Intake of iron in the full Riksmaten Adolescents sample is presented in Online Resource Table S1. Participants in the subsample had similar iron intake to those not in the subsample, except for total iron intake among girls, which was higher when expressed as mg per day (*p* = 0.038), although not when expressed as energy-standardized intake (mg per 10 MJ, *p* = 0.192).

In the subsample, the mean daily intake of total iron differed between the sexes (*p* < 0.001) and across school grades among girls (*p* = 0.013) and boys (*p* < 0.001), with the lowest intakes for both sexes in grade 5. The differences were explained by lower energy intake among girls and younger adolescents, respectively, as there were no statistically significant differences in energy-standardized iron intake. The use of dietary supplements containing iron was low; 5% among girls and 2% among boys in the iron status subsample.

Heme iron accounted for 10% of the total iron intake in girls and 14% in boys. The proportion of total iron coming from heme iron was higher among boys than girls in all age groups, with the largest difference observed in grade 11 (10% of total iron from heme iron in girls and 15% in boys, respectively, *p* < 0.001). Energy-standardized intake of heme iron was lower in girls in grades 8 and 11 compared to those in grade 5 (*p* < 0.001). Among boys, those in grade 8 had lower energy-standardized heme iron intake compared to both grade 5 (*p* = 0.020) and grade 11 (*p* < 0.001), but no statistically significant difference was observed between grade 5 and 11.

**Table 2:** Iron intake and ferritin concentration in the iron status sub-sample from Riksmaten Adolescent 2016-17

	All		Grade 5		Grade 8		Grade 11	
	Girls *n* = 579	Boys *n* = 451	Girls *n* = 164	Boys *n* = 156	Girls *n* = 222	Boys *n* = 169	Girls *n* = 193	Boys *n* = 126
Age, years, mean (SD)	14.8 (2.5)	14.4 (2.5)	11.6 (0.4)	11.6 (0.4)	14.5 (0.4)	14.5 (0.3)	17.8 (0.8)	17.8 (0.7)
Menarche, % (n) menstruating^a^	71 (407)	NA	16 (25)	NA	89 (195)	NA	98 (187)	NA
Iron supplement user, % (n)	5 (30)	2 (10)	0.6 (1)	2 (3)	4 (9)	2 (4)	10 (20)	2 (3)
Total iron intake								
mg/d, mean (SD)	7.7 (2.0)	9.6 (2.6)	7.3 (1.5)	8.1 (1.8)	7.9 (2.2)	10.5 (2.9)	7.8 (2.1)	10.2 (2.2)
mg/d, median (p25, p75)	7.4 (6.5, 8.8)	9.3 (7.9, 11.0)	7.2 (6.4, 8.2)	8.1 (6.9, 9.0)	7.5 (6.6, 9.0)	10.3 (8.6, 12.0)	7.5 (6.4, 9.1)	9.8 (8.7, 11.7)
mg/10 MJ, mean (SD)	9.6 (1.8)	9.4 (1.6)	9.5 (1.5)	9.6 (1.4)	9.7 (1.9)	9.3 (1.8)	9.5 (1.7)	9.4 (1.5)
mg/10 MJ, median (p25, p75)	9.4 (8.5,10.5)	9.3 (8.3, 10.3)	9.4 (8.4, 10.4)	9.6 (8.7, 10.4)	9.4 (8.5, 10.5)	9.0 (8.1, 10.3)	9.1 (8.4, 10.6)	9.4 (8.3. 10.3)
Non-heme iron intake								
mg/d, mean (SD)	6.9 (3.8)	8.4 (4.8)	6.4 (3.5)	7.0 (3.6)	7.4 (4.4)	9.8 (6.0)	6.7 (3.2)	8.4 (3.4)
mg/d, median (p25, p75)	6.3 (4.5, 8.2)	7.6 (5.3, 10.4)	5.8 (4.3, 7.7)	6.3 (4.5, 8.6)	6.6 (4.9, 8.4)	8.8 (5.9, 11.9)	6.2 (4.3, 8.2)	7.8 (6.2, 10.6)
mg/10 MJ, mean (SD)	8.4 (4.2)	8.1 (3.7)	8.2 (3.9)	8.2 (4.0)	9.0 (4.9)	8.4 (4.1)	8.1 (3.3)	7.6 (2.6)
mg/10 MJ, median (p25, p75)	7.7 (6.1, 9.9)	7.5 (5.8, 9.8)	7.7 (5.7, 9.7)	7.5 (5.7, 9.9)	8.1 (6.7, 10.2)	7.6 (6.0, 10.3)	7.4 (5.8, 9.8)	7.4 (6.0, 8.8)
Heme iron intake								
mg/d, mean (SD)	0.8 (0.4)	1.3 (0.6)	0.9 (0.3)	1.1 (0.6)	0.8 (0.4)	1.3 (0.6)	0.7 (0.4)	1.5 (0.4)
mg/d, median (p25, p75)	0.8 (0.5, 1.0)	1.3 (1.0, 1.7)	0.9 (0.6, 1.1)	1.1 (0.8, 1.5)	0.7 (0.5, 1.0)	1.3 (1.0, 1.6)	0.7 (0.5, 1.0)	1.5 (1.3.1.8)
mg/10 MJ, mean (SD)	1.0 (0.5)	1.3 (0.5)	1.1 (0.4)	1.3 (0.6)	0.9 (0.5)	1.2 (0.5)	0.9 (0.4)	1.4 (0.5)
mg/10 MJ, median (p25, p75)	1.0 (0.7, 1.3)	1.3 (1.0, 1.6)	1.1 (0.8, 1.4)	1.3 (0.9, 1.7)	0.9 (0.7, 1.2)	1.1 (0.9, 1.4)	0.9 (0.6, 1.3)	1.4 (1.1, 1.7)
Ferritin concentration								
µg/L, mean (SD)	31 (23)	44 (28)	32 (17)	41 (21)	28 (23)	33 (18)	31 (26)	64 (36)
µg/L, median (p25, p75)	26 (15, 39)	40 (24, 58)	30 (19, 40)	39 (26, 54)	22 (13, 35)	27 (20, 43)	27 (13, 41)	62 (42, 80)
< 15 µg/L, % (n)	23 (134)	6 (27)	10 (17)	3 (5)	30 (66)	11 (19)	26 (51)	2 (3)

^a^ Data missing for *n* = 9 girls

#### Iron status

Girls had lower ferritin concentrations than boys in all age groups (*p* < 0.001, *p* = 0.047, and *p* < 0.001 for grades 5, 8, and 11, respectively) (Table 2). The difference in mean ferritin concentrations between sexes was highest in grade 11, where boys had ferritin values that were twice as high as those of girls. ID (ferritin < 15 µg/L) was observed in 23% of the girls and 6% of the boys.

No differences in mean ferritin concentration were observed across age groups among the girls. However, ID was more prevalent in the older girls compared to those in grade 5. Notably, a much larger proportion of the older girls were menstruating compared to those in the youngest age group. Among boys, ferritin concentrations were lowest in grade 8 and highest in grade 11, and ID was more prevalent in grade 8 compared to the younger and older age groups.

#### Association between iron deficiency and background characteristics

The OR of ID was almost five times higher in girls compared to boys. The odds of ID were higher in school grades 8 (for both girls and boys) and 11 (for girls) compared to grade 5 (Online Resource Table S2). In girls, menstruation was positively associated with ID, while increased protein intake was associated with lower odds of ID. Being born outside of Sweden was associated with higher odds of ID in both girls and boys compared to being born in Sweden. No associations were observed between weight status, household education, place of residence, or E% of macronutrient intake, respectively, and ID.

### Iron intake and iron status in diets varying in climate impact

Mean energy-standardized dietary climate impact was significantly lower (*p* < 0.001) for girls than for boys, with values of 3.7 and 4.5 kg CO_2_e/10 MJ, respectively.

Linear regression analysis (unadjusted) showed that CO_2_e per 10 MJ increased by 0.08 kg for girls and 0.33 kg for boys per one mg/10 MJ increase in total iron intake (Table 3). Significant positive associations were found between the intake of heme iron, but not of non-heme iron, and dietary climate impact in both girls and boys. In girls, the mean intakes of total iron and heme iron were 5% and 180% higher, respectively, in the highest (Q4) compared to lowest (Q1) dietary climate impact groups (Q4 vs. Q1: *p* = 0.08 for total iron, *p* < 0.001 for heme iron). In boys, the mean intakes of total iron and heme iron were 21% and 138% higher, respectively, in Q4 compared to Q1 of dietary climate impact (*p* < 0.001 for both total iron and heme iron).

The proportion of girls with ID differed significantly across quartiles of dietary climate impact (*p* = 0.002) (Table 4). The OR of ID was 56% lower among girls with the highest (Q4) compared to the lowest (Q1) climate impact in the adjusted logistic regression model. A post-hoc sensitivity analysis restricted to menstruating girls (*n* = 407) showed similar results (OR 0.51, 95% CI 0.28; 0.96 for the highest vs. lowest quartile). Likewise, the association between dietary climate impact and ID was not affected by additional adjustment for iron supplement use (OR 0.42, 95% CI 0.23; 0.78 for highest vs. lowest quartile). In boys, no association was found between dietary climate impact and ID.

**Table 3:** Intakes of total, non-heme, and heme iron per 10 MJ, respectively, per sex-specific quartile of dietary climate impact (kg CO_2_e per 10 MJ), and beta coefficients with 95% CI for the linear association between energy-standardized iron intake and dietary climate impact in the iron status subsample of Riksmaten Adolescents 2016–17

		Intake					
		Total iron mg/10 MJ		Non-heme iron mg/10 MJ		Heme iron mg/10 MJ	
		Mean (SD)	Median (Q1; Q3)	Mean (SD)	Median (Q1; Q3)	Mean (SD)	Median (Q1; Q3)
Girls							
	kg CO_2_e/10 MJ						
	Q1 < 3.1	9.4 (1.7)	9.3 (8.4; 10.1)	8.7 (3.4)	8.0 (6.6; 10.4)	0.5 (0.3)	0.6 (0.3; 0.7)
	Q2 3.1–3.5	9.4 (2.1)	8.9 (5.5; 10.3)	8.8 (5.7)	7.6 (6.0; 9.8)	0.9 (0.4)	0.8 (0.7; 1.0)
	Q3 3.6–4.2	9.6 (1.8)	9.4 (8.2; 10.7)	8.3 (3.8)	7.9 (6.0; 9.5)	1.1 (0.3)	1.0 (0.9; 1.3)
	Q4 > 4.2	9.9 (1.4)	9.9 (8.9; 10.7)	7.9 (3.3)	7.3 (5.6; 9.6)	1.4 (0.3)	1.4 (1.2; 1.6)
	Linear regression (unadjusted)						
	Beta coefficient per 1 mg/10 MJ of iron intake (95% CI)	0.08 (0.03; 0.12)		-0.02 (-0.03; 0.001)		1.45 (1.35; 1.56)	
	p-value	< 0.001		0.071		< 0.001	
Boys							
	kg CO_2_e/10 MJ						
	Q1 < 3.7	8.6 (1.4)	8.5 (7.8; 9.5)	7.9 (3.9)	7.0 (5.6; 9.2)	0.8 (0.4)	0.8 (0.6; 1.0)
	Q2 3.7–4.2	9.2 (1.4)	9.1 (8.1; 10.0)	8.6 (4.0)	8.1 (6.1; 10.0)	1.1 (0.3)	1.1 (1.0; 1.3)
	Q3 4.3–5.0	9.5 (1.4)	9.5 (8.6; 10.3)	8.1 (2.8)	7.5 (6.3; 9.7)	1.4 (0.4)	1.4 (1.2; 1.6)
	Q4 > 5.0	10.4 (1.6)	10.1 (9.4; 11.1)	7.9 (4.0)	7.1 (5.8; 9.9)	1.9 (0.4)	1.8 (1.6; 2.1)
	Linear regression (unadjusted)						
	Beta coefficient per 1 mg/10 MJ of iron intake (95% CI)	0.33 (0.28; 0.39)		0.01 (-0.02; 0.04)		1.62 (1.51; 1.73)	
	p-value	< 0.001		0.511		< 0.001	

**Table 4:** Odds ratio with 95% CI of iron deficiency in relation to sex-specific quartiles of energy-standardized dietary climate impact in the iron status subsample of Riksmaten Adolescents 2016–17

			Ferritin status	Association with iron deficiency			
			*n* low/not low (% low)	Unadjusted model		Adjusted model 1^a^	
				OR	95% CI	OR	95% CI
Girls							
	kg CO_2_e/10 MJ						
		Q1 < 3.1	42/103 (29)	1.00	reference	1.00	reference
		Q2 3.1–3.5	40/104 (28)	0.94	0.57; 1.57	1.24	0.73; 2.10
		Q3 3.6–4.2	35/110 (24)	0.78	0.46; 1.32	1.03	0.67; 1.58
		Q4 > 4.2	17/128 (12)	0.33	0.18; 0.61	0.44	0.24; 0.81
Boys							
	kg CO_2_e/10 MJ						
		Q1 < 3.7	11/112 (10)	1.00	reference	1.00	reference
		Q2 3.7–4.2	5/114 (4)	0.42	0.14; 1.25	0.54	0.17; 1.79
		Q3 4.3–5.0	4/112 (4)	0.34	0.10; 1.10	0.51	0.15; 1.72
		Q4 > 5.0	7/113 (6)	0.61	0.23; 1.63	0.93	0.29; 2.95

^a^ Model adjusted for school grade, country of birth (Sweden/outside Sweden), menstruation (yes/no) among girls, and includes school as cluster variable

### Dietary sources of iron

**Fig. 1:**
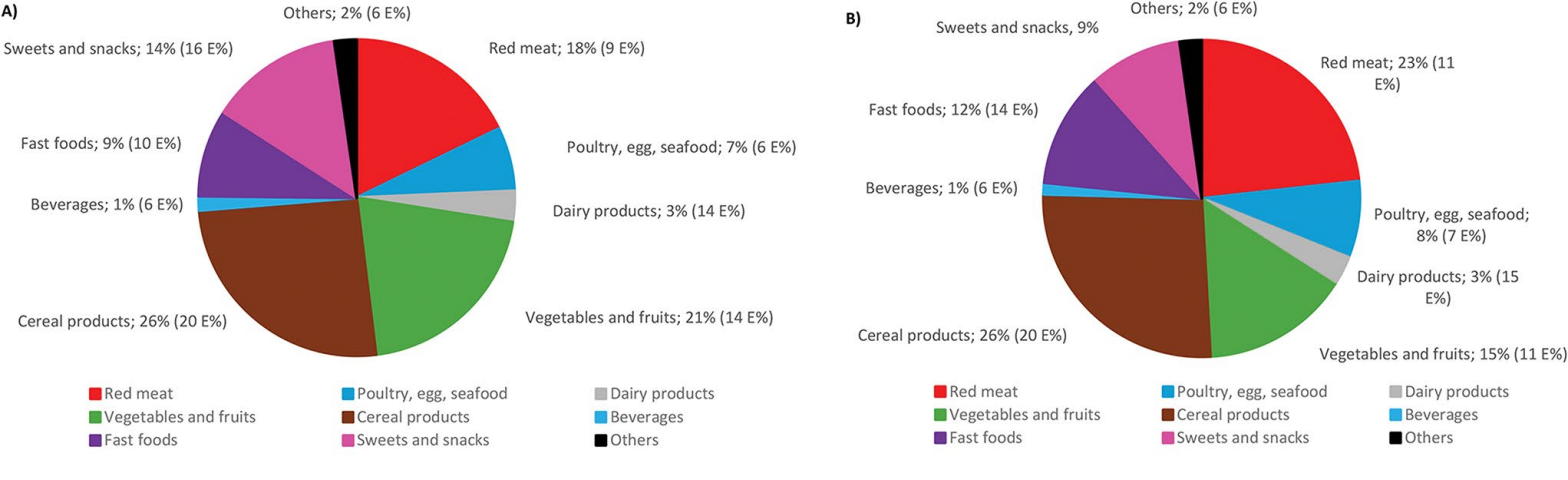
Contribution of mean total iron (and energy) intake per food group in girls (**A**) and boys (**B**) in the full sample of Riksmaten Adolescents. The underlying data are found in Online Resource Table S3

Figure 1 illustrates the contribution of total iron by food group for girls and boys. The food groups also include composite dishes. For both sexes, ‘cereal products’, ‘red meat’ and ‘vegetables and fruits’ were the main sources of iron, together accounting for 60% of the total iron intake. Bread (10%), pasta (6%) and ‘breakfast cereals’ (6%) contributed the largest portion of total iron from cereal products. The intake of total iron from red meat primarily came from unprocessed (13%) and processed (5%) red meat products and dishes. ‘Vegetables, roots, and pulses’ (6%), potatoes (5%), and ‘fruits and berries’ (4%) accounted for the largest contribution of total iron from ‘vegetables and fruits’. Detailed results for the contribution of total iron from specific food groups are presented in Online Resource Table S3.

‘Red meat’, ‘fast foods’, and ‘poultry, egg, and seafood’ were the main sources of heme iron, accounting for 93% of the intake of heme iron in all participants (Online Resource Table S3). Within these aggregated food groups, the major sources of heme iron were red unprocessed meat (53%), red processed meat (15%), poultry (7%), hamburgers (6%), and ‘offal and blood products’ (5%), with small differences observed between the sexes.

### Association between iron status and iron intake

Odds ratios for ID by intake of iron (total, heme and non-heme) are presented in Table 5. Girls in the highest quartile of heme iron intake (> 1.00 mg/d) had 50% lower odds (OR 0.50, 95% CI 0.30; 0.84) of ID compared to girls in the lowest quartile (Q1 ≤ 0.53 mg/d) in the adjusted model (Table 5). However, no associations were observed between quartiles of total iron intake and ID. Additional adjustment for the use of iron supplements did not alter these results. In boys, no associations were observed between quartiles of iron intake and ID.

**Table 5:** Odds ratios with 95% CI for iron deficiency by iron intake among adolescents in the iron status subsample of Riksmaten Adolescents 2016-17

		*n* low/not low (% low)	Unadjusted model		Adjusted model 1^a^	
			OR	95% CI	OR	95% CI
**Girls**						
Iron intake, mg/d						
	Q1 ≤ 6.5	36/108 (25)	1.00	reference	1.00	reference
	Q2 6.5–7.4	33/112 (23)	0.88	0.51; 1.52	0.82	0.51; 1.33
	Q3 7.4–8.8	29/117 (20)	0.74	0.43; 1.29	0.81	0.45; 1.46
	Q4 > 8.8	36/108 (25)	1.00	0.59; 1.70	0.85	0.53; 1.37
Non-heme iron intake, mg/d						
	Q1 ≤ 4.4	28/117 (19)	1.00	reference	1.00	reference
	Q2 4.5–6.2	43/101 (30)	1.78	1.03; 3.07	1.65	1.05; 2.60
	Q3 6.3–8.2	28/118 (19)	0.99	0.55; 1.78	0.98	0.55; 1.74
	Q4 > 8.2	35/109 (24)	1.34	0.77; 2.35	1.21	0.72; 2.01
Heme iron intake, mg/d						
	Q1 ≤ 0.53	43/102 (30)	1.00	reference	1.00	reference
	Q2 0.53–0.75	39/105 (27)	0.88	0.53; 1.47	1.01	0.60; 1.70
	Q3 0.75–1.00	31/114 (21)	0.65	0.38; 1.10	0.79	0.48; 1.31
	Q4 >1.00	21/124 (14)	0.40	0.22; 0.72	0.50	0.30; 0.84
**Boys**						
Iron intake, mg/d						
	Q1 ≤ 7.9	5/108 (4)	1.00	reference	1.00	reference
	Q2 7.9–9.3	6/106 (6)	1.22	0.36; 4.13	1.42	0.30; 6.67
	Q3 9.3–11.0	8/105 (7)	1.65	0.52; 5.19	1.61	0.45; 5.79
	Q4 > 11.0	8/105 (7)	1.65	0.52; 5.19	1.34	0.47; 3.81
Non-heme iron intake, mg/d						
	Q1 ≤ 5.3	6/107 (5)	1.00	reference	1.00	reference
	Q2 5.4–7.6	4/109 (4)	0.65	0.18; 2.38	0.59	0.21; 1.66
	Q3 7.7–10.3	9/103 (8)	1.56	0.54; 4.53	1.30	0.51; 3.36
	Q4 > 10.3	8/105 (7)	1.36	0.46; 4.05	1.07	0.41; 2.81
Heme iron intake, mg/d						
	Q1 ≤ 0.95	9/103 (8)	1.00	reference	1.00	reference
	Q2 0.95–1.31	6/107 (5)	0.64	0.22; 1.87	0.76	0.16; 3.68
	Q3 1.31–1.66	7/107 (6)	0.75	0.27; 2.08	1.03	0.32; 3.28
	Q4 > 1.66	5/107 (4)	0.53	0.17; 1.65	0.68	0.22; 2.08

^a^ Model adjusted for school grade, country of birth (Sweden/outside Sweden), menstruation (yes/no) among girls, and includes school as cluster variable

### Association between iron status and food intake

In logistic regression analysis, girls in the highest quartile of red meat intake (> 92 g/d) had 39% lower odds (OR 0.61, 95% CI 0.37; 0.99) of ID compared to girls in the lowest quartile of intake (< 57 g/d) in the most adjusted model (Online Resource Table S4). This relationship remained robust in the sensitivity analysis after additional adjustment for iron supplement use (OR 0.58, 95% CI 0.36; 0.95 for highest vs. lowest quartile). Boys in the highest quartile of dairy intake (> 710 g/d) had significantly higher odds (OR 4.20, 95% CI 1.63; 10.83) of ID compared to boys in the lowest quartile of intake (Q1 < 285 g/d) in the most adjusted model (Online Resource Table S5). No other associations with the assessed food groups (red meat, cereal products, fruits and vegetables, dairy products, sweet foods, sugar-sweetened beverages) were evident. Additional data on food intake levels in girls and boys, and across different age groups within the subsample, are shown in Online Resource Table S6.

Few participants reported omitting meat from their diet; 31 girls and three boys. The prevalence of ID among girls who omitted meat was 35%, compared to 22% among girls who reported being meat consumers. However, the difference in prevalence of ID between these groups was not statistically significant.

## Discussion

This study assessed iron intake and the prevalence of ID among Swedish adolescents with varying dietary climate impact. Adolescents with higher dietary climate impact had higher intakes of total iron and heme iron, but not non-heme iron. The odds of ID were lower among girls with the highest compared to the lowest dietary climate impact, while no association was found between dietary climate impact and ID in boys. This study revealed large sex differences; boys had higher intakes of both iron and red meat, higher dietary climate impact, and lower prevalence of ID compared to the girls.

### Iron intake and status in Swedish adolescents

In this study, large differences were observed between the sexes; 23% of the girls and 6% of the boys were classified as iron deficient, and the girls had lower ferritin concentrations than the boys in all age groups. Compared to the girls, the boys had, on average, 25% higher intake of total iron per day and a higher proportion from heme iron in all age groups. The mean intake levels of iron for girls (7.7 mg/d) were below dietary reference values for average requirements (9–10 mg/d), whereas the intake levels for boys (9.6 mg/d) exceeded the average requirements (9 mg/d) but not the recommended intake (11 mg/d) [6]. This suggests that boys may have some opportunity to reduce their intake of iron-rich foods, such as red meat, and the related climate impact, without risking ID.

### Risk groups for iron deficiency

The risk of ID depends on iron requirements, which vary between girls and boys, as well as among different age groups of adolescents. In this study, ID was most prevalent among adolescents aged 14–15 years (30% of girls, 11% of boys) and among girls aged 17–18 years (26%). These results reflect the elevated iron requirement in girls to support growth and menstruation, and in boys for the pubertal growth spurt [8]. Boys, on average, mature later than girls and have shorter duration of their adolescent growth period, which is reflected in low prevalence of ID (2–3%) in 11–12- and 17–18-year-old boys in the current study. In contrast, 10% of the girls were iron deficient even in the youngest age group studied.

Girls with menstruation were indicated as a risk group for ID. Notably, the odds of ID were almost five times higher in girls compared to boys, and three times higher in menstruating girls compared to those not menstruating. Being born outside of Sweden was indicated as an additional risk factor for ID in both girls and boys; however, potential explanations for this observation could not be further explored in these data. Extra attention should be given to ensure adequate iron intake and status among these risk groups.

Further research is needed to investigate whether the increased risk of ID among individuals born outside of Sweden can be confirmed in other studies and in other age groups, as well as to explore underlying factors that may explain the increased risk of ID in this population.

### Increased risk of iron deficiency in low-climate diets?

This study found a significant positive association between dietary climate impact and the intake of total and heme iron in both girls and boys. However, no such associations were observed for the intake of non-heme iron, highlighting the differences between the types of iron. These results indicate that diets with a lower climate impact in Swedish adolescents contain less total iron and, in particular, less heme iron, which has higher bioavailability compared to non-heme iron. To understand the health implications of this, it is necessary to evaluate whether the climate impact of diet also affects iron status and the risk of ID. Notably, the proportion of girls with ID differed across quartiles of dietary climate impact, while no association between dietary climate impact and ID was found in boys.

Boys had 22% higher energy-standardized dietary climate impact compared to girls, largely driven by higher meat intake. In fact, the highest quartile of red meat intake among the girls corresponded to the lowest quartile of intake among the boys. Our results highlight the importance of considering the risk of ID in interventions for climate-adapted diets, particularly for risk groups of low iron status, such as menstruating girls. However, the concern about ID is less justified among most boys, where efforts to reduce dietary climate impact are most needed. It should be noted that the mean dietary climate impact of both girls and boys in this study exceeds proposed per capita climate targets for the food system [2] by more than two-fold. Thus, for most Swedish adolescents, large reductions in dietary climate impact would be required to achieve this goal, which may have further implications for nutrient intake and status.

### Role of specific food groups and types of iron

In this study, heme iron accounted for 10% and 14% of total iron intake in girls and boys, respectively. Both animal-based and plant-based food groups were identified as important sources of total iron intake, whereas the intake of heme iron is restricted to animal-based foods. For a correct interpretation of these results, it should be borne in mind that food categorization was based on the main ingredient in composite dishes. For example, a vegetable soup containing small amounts of meat was classified in the category of vegetables, which explains the contribution of heme iron from some plant-based food categories.

The bioavailability of heme iron is generally higher (about 25%) compared to non-heme iron, for which the absorption is more affected by dietary compounds and iron stores [8]. In this study, the odds of ID were halved in girls with the highest intake of heme iron compared to those with the lowest intake. In contrast, no association was observed between the intake of total iron and ID. Even in this perspective, the results differed between the sexes, as no associations were observed between quartiles of iron intake (total, heme iron, non-heme iron) and ID among the boys. These findings suggest that the intake of heme iron may indeed influence the risk of ID in girls, while, apart from this, other factors seem to have a greater impact on the risk of ID than iron intake.

Red meat’s high content of heme iron, with high bioavailability, and its simultaneously high climate impact per kilogram, compared to other food groups, is a main reason for concern of a goal conflict between nutrition and environmental perspectives in the pursuit of sustainable diets. Therefore, it is of interest to investigate how the risk of ID is affected by dietary composition. In this study, lower intake of red meat in girls and higher intake of dairy products in boys were associated with higher odds of ID. The odds of ID were 39% lower in girls with the highest compared to the lowest intake of red meat. Notably, only the girls in the lowest quartile of red meat intake had intake levels below the upper limit of 350 g per week (50 g/d) recommended by the Nordic Nutrition Recommendations 2023 [6]. This highlights that Swedish adolescents, especially boys, who consume more red meat than girls, are eating more red meat than currently recommended from an overall health perspective. The potential for reducing the risk of ID through increased intake of red meat therefore needs to be weighed against other health aspects and environmental considerations.

Boys in the highest quartile of dairy intake had four times higher odds of ID compared to those in the lowest quartile. Calcium is known to reduce both heme and non-heme iron absorption [23], which may partly explain the observed association. Alternatively, boys with high dairy intake may have a diet with lower iron content compared to those with lower intake of dairy products. Higher milk consumption has previously been associated with an increased risk of ID in Norwegian girls [29]. In this study, the association was found only in boys, which may be due to their higher intake of dairy products compared to girls. Dairy intake was highest among boys in grade 8, representing an age group with high pubertal growth and elevated iron needs, which may also have contributed to the observed association with higher risk of ID in boys in this study.

### Results in relation to previous findings

According to a recent review [13], iron is the micronutrient most frequently studied in relation to dietary environmental impact. However, none of the studies identified in the review assessed the relationship between dietary environmental impact and iron status, which makes it difficult to compare our results with previous findings. The review identified six studies that analyzed iron intake in relation to dietary climate impact, three of which showed significantly lower iron intake in diets with lower climate impact, while no statistically significant differences were observed in the remaining three studies. While the overall results from the review suggest that diets aiming to reduce environmental impact may lower micronutrient intake, the results for iron differed between studies and by methodological approach [13]. A previous review found associations between increased iron intake and a reduction in dietary climate impact in 20 out of 38 diet studies assessed [5].

Other studies have assessed iron intake and status in relation to animal-based food intake but have not considered the environmental impact of diets. For example, a randomized controlled trial in Finland studied the effect of replacing animal-source proteins with plant-based proteins [30] and found that iron intake was higher in diets based on 30% animal-based protein compared to diets with higher proportions (50% or 70%), whereas no significant differences were observed in biomarkers of iron status (Hb, ferritin, transferrin receptor). In contrast, a meta-analysis of 24 cross-sectional studies showed that adult vegetarians had significantly lower serum ferritin levels compared to non-vegetarians [31]. Moreover, a study of European adolescents found no association between iron intake and biomarkers of iron status (ferritin, hematocrit, Hb, soluble transferrin receptor), apart from the concentration of red blood cells, which was negatively associated with total iron intake, heme iron, and non-heme iron [32].

In summary, there is a lack of knowledge about how iron status may be affected by a transition to more environmentally friendly diets, and existing studies show conflicting results. The complex regulation of the body’s iron stores means that changes in iron intake may not ultimately affect iron status, which is why more research is needed to explore the risk of ID based on biomarkers in relation to the environmental impact of diets.

### Strengths and limitations

Strengths of this study include the availability of both dietary iron intake data and iron status based on plasma ferritin concentration. In addition, the intake of iron was decomposed into heme iron and non-heme iron, which enables the study of differences between types of iron as well as between iron intake and iron status. This approach provides a better understanding of the risk of ID in climate-adapted diets. Another strength is the focus on adolescents, a critical group due to their elevated iron needs. The use of a national survey, where detailed information on food and beverage intake was collected through a 24-hour recall method and where reported energy intake was judged to be plausible [15], further enhances the study´s reliability.

The main limitation of the study is the cross-sectional design, which hinders conclusions about causality. Furthermore, although blood sample data are available for more than 1000 participants in Riksmaten Adolescents 2016–17, the sample size still limits the possibility of performing more detailed subgroup analyses. However, the participants with available iron status data were shown to be similar to those without blood sample data, and the full Riksmaten Adolescents 2016–17 dataset has been shown to be nationally representative of Swedish adolescents [17]. Additionally, a two-day food record may not correctly capture habitual food intake. To overcome this, dietary intake was transformed from current intake to long-term intake [2, 27]; however, this could still attenuate the findings. Uncertainty due to recall-bias and dietary misreporting, such as underreporting of unhealthy foods, is a known challenge in self-reported dietary data [33] and may have also affected the estimated food intake levels and subsequent results for iron intake and dietary climate impact. The low prevalence of ID in boys makes the statistical analyses uncertain, particularly the logistic regression analysis, and hampers the ability to draw conclusions. On the other hand, since ID is more frequent among girls, where the analyses may be more robust due to higher numbers, this study still contributes valuable knowledge. Another limitation is the low variation in dietary climate impact in this sample, which may have contributed to the lack of an inverse dose-response relationship with ID. Uncertainties also exist in climate impact assessments of foods and diets [34]. In this study, efforts were made to reduce the uncertainty by using LCA data from a database that is annually updated and by applying LCA data representative of Swedish consumption to capture major variations in impact between production systems. The nutritional focus of this study is limited to iron, without any consideration of other nutrients. Previous studies have found that diets with lower climate impact often have lower intakes of saturated fat and salt, whereas the intake of several micronutrients may be compromised, including vitamin D, A, B12, calcium, iodine, and zinc [5, 13]. For a more holistic sustainability perspective, the scope of this research needs to be broadened to account for additional environmental and sustainability indicators.

### Future research

Longitudinal studies based on repeated observations over longer time periods are needed to further investigate potential explanations for the observed differences in ID risk across population groups. Studies that include food intake data and blood samples from a large number of individuals are necessary to allow for detailed subgroup analyses to gain more knowledge about specific population groups and characteristics associated with more and less sustainable diets. More studies are warranted, especially focusing on identified risk groups for ID in the population (e.g., menstruating girls and individuals born outside of Sweden). To support the development of guidelines for sustainable diets, more studies are needed that examine the combined environmental impact and nutrient adequacy of diets, assessed based on both nutrient intake and nutrient status. Future studies should be based on a broad sustainability perspective, considering additional nutrients and environmental indicators beyond those analyzed in this study.

## Conclusions

This study contributes to a better understanding of the potential trade-offs between nutritional and environmental aspects of sustainable diets, and more specifically, how adequate iron status can be ensured in future climate-smart diets. The findings indicate that girls with a more climate-friendly diet and lower intake of red meat/heme iron have higher prevalence of ID compared to girls with a higher dietary climate impact. Compared to girls, boys had a higher dietary climate impact and a lower prevalence of ID. These results highlight the importance of considering risk groups for ID, such as menstruating girls, in the transition to a more plant-based diet with lower climate impact.

## Electronic supplementary material

Below is the link to the electronic supplementary material.

Supplementary Material 1
