# Supplementary material for: Iron intake and iron status of Swedish adolescents with diets of varying climate impact
Source: Eur J Nutr. 2025 Feb 15;64(2):93. doi: 10.1007/s00394-024-03572-y (PMC11829901; doi:10.1007/s00394-024-03572-y)
Supplement: Supplementary file 1 — Supplementary Material 1 [file 394_2024_3572_MOESM1_ESM.docx]

# **Supplementary Materials: Iron intake and iron status of Swedish adolescents with diets of varying climate impact**

Elinor Hallström^1,2ǂ*^, Josefin Edwall Löfvenborg^3ǂ^, Lotta Moreaus^3^, Agneta Sjöberg^4^, Anna Winkvist^5^, Anna Karin Lindroos^3,5^

^1^Nutrition, Sustainability and Health Promotion Group, National Food Institute, Technical University of Denmark, Kgs Lyngby, Denmark

^2^Department of Food and Agriculture, Research Institutes of Sweden (RISE), Lund, Sweden

^3^Division for Risk and Benefit Assessment, Swedish Food Agency, Uppsala, Sweden

^4^Department of Food and Nutrition and Sport Science, University of Gothenburg, Gothenburg, Sweden

^5^Department of Internal Medicine and Clinical Nutrition, the Sahlgrenska Academy, University of Gothenburg, Gothenburg, Sweden

^ǂ^ Shared first author

*Corresponding author: eemha@dtu.dk

European Journal of Nutrition

|  |  | **All** | | **Grade 5** | | **Grade 8** | | **Grade 11** | |
| --- | --- | --- | --- | --- | --- | --- | --- | --- | --- |
|  |  | **Girls** n=1710 | **Boys** n=1389 | **Girls** n=559 | **Boys** n=490 | **Girls** n=574 | **Boys** n=476 | **Girls** n=577 | **Boys** n=423 |
| Age, years, mean (SD) | | 14.6 (2.6) | 14.5 (2.6) | 11.5 (0.4) | 11.5 (0.4) | 14.5 (0.4) | 14.5 (0.4) | 17.7 (0.6) | 17.7 (0.6) |
| Menarche, n (%) mentruating^a^ | | 1146 (68) | NA | 71 (13) | NA | 513 (90) | NA | 562 (98) | NA |
| Iron supplement user, % (n) | | 2 (37) | 0.9 (12) | 0.5 (3) | 0.6 (3) | 2 (10) | 0.8 (4) | 4 (24) | 1 (5) |
| Total iron intake | |  |  |  |  |  |  |  |  |
|  | mg/d, mean (SD) | 7.6 (1.9) | 9.4 (2.8) | 7.2 (1.6) | 8.1 (2.1) | 7.6 (2.1) | 9.8 (2.8) | 7.8 (2.0) | 10.4 (2.8) |
|  | mg/d, median (Q1, Q3) | 7.4 (6.3, 8.7) | 9.0 (7.5, 10.9) | 7.1 (6.2, 8.2) | 8.0 (6.7, 9.1) | 7.4 (6.2, 8.8) | 9.6 (7.9, 11.3) | 7.7 (6.5, 9.0) | 9.9 (8.6, 12.0) |
|  | mg/10 MJ, mean (SD) | 9.5 (1.7) | 9.5 (1.7) | 9.4 (1.5) | 9.6 (1.5) | 9.6 (1.7) | 9.3 (1.7) | 9.5 (1.8) | 9.6 (1.8) |
|  | mg/10 MJ, median (Q1, Q3) | 9.3 (8.4, 10.4) | 9.4 (8.4, 10.4) | 9.2 (8.4, 10.2) | 9.6 (8.6, 10.5) | 9.4 (8.5, 10.5) | 9.0 (8.2, 10.2) | 9.3 (8.2, 10.6) | 9.5 (8.4, 10.4) |
| Non-heme iron intake | |  |  |  |  |  |  |  |  |
|  | mg/d, mean (SD) | 6.7 (3.4) | 8.3 (5.1) | 6.3 (3.1) | 7.1 (4.5) | 6.7 (3.7) | 9.0 (5.6) | 7.0 (3.3) | 8.9 (5.0) |
|  | mg/d, median (Q1, Q3) | 6.2 (4.4, 8.2) | 7.4 (5.1, 10.4) | 5.6 (4.2, 7.9) | 6.5 (4.5, 8.8) | 6.1 (4.3, 8.0) | 7.9 (5.3, 11.6) | 6.5 (4.8, 8.6) | 8.1 (5.7, 11.3) |
|  | mg/10 MJ, mean (SD) | 8.3 (3.6) | 8.2 (4.0) | 8.0 (3.5) | 8.4 (4.4) | 8.3 (3.9) | 8.2 (3.9) | 8.4 (3.5) | 7.9 (3.5) |
|  | mg/10 MJ, median (Q1, Q3) | 7.8 (6.0, 9.8) | 7.6 (5.8, 10.0) | 7.4 (5.6, 9.5) | 7.8 (5.8, 10.1) | 7.8 (6.2, 9.8) | 7.4 (5.7, 10.1) | 8.0 (6.2, 10.1) | 7.6 (5.9, 9.7) |
| Heme iron intake | |  |  |  |  |  |  |  |  |
|  | mg/d, mean (SD) | 0.8 (0.4) | 1.3 (0.5) | 0.9 (0.3) | 1.1 (0.6) | 0.8 (0.4) | 1.2 (0.5) | 0.8 (0.4) | 1.5 (0.5) |
|  | mg/d, median (Q1, Q3) | 0.8 (0.5, 1.0) | 1.3 (0.9, 1.6) | 0.9 (0.7, 1.1) | 1.1 (0.8, 1.5) | 0.7 (0.5, 1.0) | 1.2 (0.9, 1.5) | 0.7 (0.5, 1.0) | 1.5 (1.3, 1.8) |
|  | mg/10 MJ, mean (SD) | 1.0 (0.5) | 1.3 (0.5) | 1.2 (0.4) | 1.4 (0.6) | 1.0 (0.5) | 1.2 (0.5) | 0.9 (0.5) | 1.5 (0.5) |
|  | mg/10 MJ, median (Q1, Q3) | 1.0 (0.7, 1.3) | 1.3 (1.0, 1.7) | 1.1 (0.9, 1.4) | 1.4 (0.9, 1.7) | 0.9 (0.6, 1.2) | 1.2 (0.9, 1.4) | 0.9 (0.6, 1.2) | 1.4 (1.1, 1.7) |
| ^a^ Data missing for n=16 girls. | |  |  |  |  |  |  |  |  |

**Table S1.** Iron intake in the full sample of Riksmaten Adolescents 2016–17.

**Table S2** Unadjusted odds ratios (OR) with 95% confidence intervals (CI) of iron deficiency (ferritin <15 µg/L) by background characteristics in the iron status subsample of Riksmaten Adolescents 2016-17.

|  |  | **Girls n=579** | | **Boys n=451** | |
| --- | --- | --- | --- | --- | --- |
|  |  |  | |  | |
|  |  | Crude OR | 95% CI | Crude OR | 95% CI |
|  |  |  |  |  |  |
| Grade | Grade 8 vs. grade 5 | 3.66 | 2.05; 6.53 | 3.83 | 1.39; 10.51 |
|  | Grade 11 vs. grade 5 | 3.11 | 1.71; 5.63 | 0.74 | 0.17; 3.14 |
|  |  |  |  |  |  |
| Menarche^a^ | Menstruating vs. not | 3.21 | 1.88; 5.48 | NA | --- |
|  |  |  |  |  |  |
| Weight status | Overweight/obese vs. normal weight/underweight | 0.96 | 0.59; 1.57 | 0.45 | 0.13; 1.53 |
|  |  |  |  |  |  |
| Household education^b^ | >12 years vs. ≤12 years | 0.97 | 0.64; 1.45 | 0.63 | 0.28; 1.42 |
|  |  |  |  |  |  |
| Country of birth^c^ | Born outside Sweden vs. in Sweden | 3.71 | 2.14; 6.44 | 2.68 | 1.08; 6.66 |
|  |  |  |  |  |  |
| Place of residence | Urban vs. rural | 1.20 | 0.80; 1.80 | 0.47 | 0.21; 1.02 |
|  |  |  |  |  |  |
| Energy intake | Per 1 MJ increase in energy intake | 1.03 | 0.92; 1.15 | 1.12 | 0.98; 1.29 |
|  | Per 1 E% increase in protein intake | 0.92 | 0.85; 1.00 | 0.95 | 0.82; 1.09 |
|  | Per 1 E% increase in fat intake | 1.00 | 0.96; 1.05 | 0.93 | 0.85; 1.03 |
|  | Per 1 E% increase in carbohydrate intake | 1.02 | 0.98; 1.06 | 1.07 | 1.00; 1.15 |
| ^a^ Data missing for n=9 in the subsample. ^b^ Data missing for n=26 girls and n=24 boys. ^c^ Data missing for n=2 girls. | | | | | |

**Table S3.** Intake of energy, total iron and heme iron per food group for all participants, girls and boys, in Riksmaten Adolescents 2016–17.

|  | **All participants (n=3099)** | | | **Girls (n=1710)** | | | **Boys (n=1389)** | | |
| --- | --- | --- | --- | --- | --- | --- | --- | --- | --- |
| **FOOD GROUPS** | **Energy**  **kJ (%)** | **Total iron mg (%)** | **Heme iron mg (%)** | **Energy**  **kJ (%)** | **Total iron mg (%)** | **Heme iron. mg (%)** | **Energy**  **kJ (%)** | **Total iron mg (%)** | **Heme iron mg (%)** |
| **RED MEAT** | **867 (10%)** | **1.72 (19%)** | **0.75 (73%)** | **691 (9%)** | **1.35 (18%)** | **0.58 (73%)** | **1084 (11%)** | **2.19 (23%)** | **0.95 (73%)** |
| Red unprocessed meat | 519 (6%) | 1.19 (13%) | 0.54 (53%) | 413 (5%) | 0.95 (12%) | 0.43 (54%) | 649 (7%) | 1.50 (16%) | 0.69 (52%) |
| Processed red meat | 338 (4%) | 0.40 (5%) | 0.15 (15%) | 273 (3%) | 0.33 (4%) | 0.12 (16%) | 417 (4%) | 0.49 (5%) | 0.18 (14%) |
| Offal and blood products | 10.2 (0%) | 0.13 (1%) | 0.05 (5%) | 4.98 (0%) | 0.07 (1%) | 0.03 (4%) | 17 (0%) | 0.20 (2%) | 0.08 (6%) |
| **POULTRY, EGG, SEAFOOD** | **546 (6%)** | **0.60 (7%)** | **0.10 (9%)** | **458 (6%)** | **0.49 (7%)** | **0.08 (10%)** | **654 (7%)** | **0.74 (8%)** | **0.12 (9%)** |
| Poultry | 252 (3%) | 0.29 (3%) | 0.07 (7%) | 208 (3%) | 0.23 (3%) | 0.06 (7%) | 305 (3%) | 0.36 (4%) | 0.09 (7%) |
| Egg | 80.8 (1%) | 0.19 (2%) | 0.00 (0%) | 66 (1%) | 0.16 (2%) | 0.00 (0%) | 99 (1%) | 0.23 (2%) | 0.00 (0%) |
| Seafood | 213 (2%) | 0.12 (1%) | 0.03 (3%) | 184 (2%) | 0.10 (1%) | 0.02 (3%) | 250 (3%) | 0.14 (2%) | 0.03 (3%) |
| **DAIRY PRODUCTS** | **1264 (14%)** | **0.26 (3%)** | **0.00 (0%)** | **1090 (14%)** | **0.24 (3%)** | **0.00 (0%)** | **1479 (15%)** | **0.28 (3%)** | **0.00 (0%)** |
| Milk, yoghurt, cream, pancakes | 1060 (12%) | 0.23 (3%) | 0.00 (0%) | 902 (11%) | 0.22 (3%) | 0.00 (0%) | 1255 (13%) | 0.26 (3%) | 0.00 (0%) |
| Cheese | 205 (2%) | 0.02 (0%) | 0.00 (0%) | 188 (2%) | 0.02 (0%) | 0.00 (0%) | 225 (2%) | 0.03 (0%) | 0.00 (0%) |
| **VEGETABLES AND FRUITS** | **1093 (12%)** | **1.49 (17%)** | **0.04 (4%)** | **1109 (14%)** | **1.56 (21%)** | **0.04 (5%)** | **1074 (11%)** | **1.42 (15%)** | **0.04 (3%)** |
| Vegetables, roots, pulses | 283 (3%) | 0.55 (6%) | 0.03 (3%) | 317 (4%) | 0.59 (8%) | 0.03 (4%) | 241 (2%) | 0.49 (5%) | 0.03 (3%) |
| Fruits and berries | 197 (2%) | 0.34 (4%) | 0.00 (0%) | 222 (3%) | 0.39 (5%) | 0.00 (0%) | 166 (2%) | 0.29 (3%) | 0.00 (0%) |
| Potatoes | 523 (6%) | 0.46 (5%) | 0.01 (1%) | 454 (6%) | 0.39 (5%) | 0.01 (1%) | 607 (6%) | 0.55 (6%) | 0.01 (1%) |
| Nuts and seeds | 45.4 (1%) | 0.06 (1%) | 0.00 (0%) | 54.0 (1%) | 0.08 (1%) | 0.00 (0%) | 34.8 (0%) | 0.04 (0%) | 0.00 (0%) |
| Plant-based dairy subsitutes | 16.0 (0%) | 0.02 (0%) | 0.00 (0%) | 21.2 (0%) | 0.02 (0%) | 0.00 (0%) | 9.53 (0%) | 0.01 (0%) | 0.00 (0%) |
| Plant-based meat substitutes | 29.6 (0%) | 0.06 (1%) | 0.00 (0%) | 40.9 (1%) | 0.08 (1%) | 0.00 (0%) | 15.6 (0%) | 0.03 (0%) | 0.00 (0%) |
| **CEREAL PRODUCTS** | **1743 (20%)** | **2.18 (24%)** | **0.03 (3%)** | **1582 (20%)** | **1.94 (26%)** | **0.02 (3%)** | **1942 (20%)** | **2.48 (26%)** | **0.04 (3%)** |
| Bread | 782 (9%) | 0.88 (10%) | 0.00 (0%) | 765 (10%) | 0.85 (11%) | 0.00 (0%) | 803 (8%) | 0.90 (10%) | 0.00 (0%) |
| Breakfast cereals | 164 (2%) | 0.51 (6%) | 0.00 (0%) | 135 (2%) | 0.40 (5%) | 0.00 (0%) | 201 (2%) | 0.64 (7%) | 0.00 (0%) |
| Grains | 20.6 (0%) | 0.04 (0%) | 0.00 (0%) | 23.2 (0%) | 0.05 (1%) | 0.00 (0%) | 17.4 (0%) | 0.04 (0%) | 0.00 (0%) |
| Porrige | 91.9 (1%) | 0.14 (2%) | 0.00 (0%) | 80.3 (1%) | 0.12 (2%) | 0.00 (0%) | 106 (1%) | 0.17 (2%) | 0.00 (0%) |
| Pasta | 473 (5%) | 0.49 (6%) | 0.03 (3%) | 393 (5%) | 0.42 (6%) | 0.02 (3%) | 571 (6%) | 0.59 (6%) | 0.04 (3%) |
| Rice | 212 (2%) | 0.12 (1%) | 0.00 (0%) | 186 (2%) | 0.11 (1%) | 0.00 (0%) | 243 (2%) | 0.14 (1%) | 0.00 (0%) |
| **BEVERAGES** | **525 (6%)** | **0.11 (1%)** | **0.00 (0%)** | **453 (6%)** | **0.11 (1%)** | **0.00 (0%)** | **613 (6%)** | **0.11 (1%)** | **0.00 (0%)** |
| Juice | 130 (1%) | 0.07 (1%) | 0.00 (0%) | 125 (2%) | 0.06 (1%) | 0.00 (0%) | 135 (1%) | 0.07 (1%) | 0.00 (0%) |
| Soda, cordial, coffe, tea etc. | 351 (4%) | 0.03 (0%) | 0.00 (0%) | 291 (4%) | 0.02 (0%) | 0.00 (0%) | 426 (4%) | 0.03 (0%) | 0.00 (0%) |
| Alcoholic beverages | 43.9 (0%) | 0.02 (0%) | 0.00 (0%) | 37.6 (0%) | 0.02 (0%) | 0.00 (0%) | 51.8 (1%) | 0.01 (0%) | 0.00 (0%) |
| **FAST FOOD** | **1075 (12%)** | **0.87 (10%)** | **0.10 (10%)** | **833 (10%)** | **0.67 (9%)** | **0.07 (8%)** | **1373 (14%)** | **1.11 (12%)** | **0.14 (11%)** |
| Hamburgers | 190 (2%) | 0.23 (3%) | 0.06 (6%) | 127 (2%) | 0.17 (2%) | 0.04 (4%) | 268 (3%) | 0.31 (3%) | 0.09 (7%) |
| Pizza, pie, sandwich | 885 (10%) | 0.64 (7%) | 0.04 (4%) | 706 (9%) | 0.51 (7%) | 0.03 (4%) | 1105 (11%) | 0.80 (8%) | 0.05 (4%) |
| **SWEETS AND SNACKS** | **1223 (14%)** | **1.1 (13%)** | **0 (0%)** | **1260 (16%)** | **1.03 (14%)** | **0.00 (0%)** | **1177 (12%)** | **0.9 (9%)** | **0.00 (0%)** |
| Sweet bread, cakes, cookies | 302 (3%) | 0.22 (2%) | 0.00 (0%) | 342 (4%) | 0.25 (3%) | 0.00 (0%) | 252 (3%) | 0.17 (2%) | 0.00 (0%) |
| Desserts | 50.0 (1%) | 0.03 (0%) | 0.00 (0%) | 52.8 (1%) | 0.03 (0%) | 0.00 (0%) | 46.7 (0%) | 0.03 (0%) | 0.00 (0%) |
| Sugar, syrup, honey | 76.7 (1%) | 0.03 (0%) | 0.00 (0%) | 68.4 (1%) | 0.03 (0%) | 0.00 (0%) | 87.0 (1%) | 0.04 (0%) | 0.00 (0%) |
| Ice cream | 71.5 (1%) | 0.02 (0%) | 0.00 (0%) | 74.5 (1%) | 0.02 (0%) | 0.00 (0%) | 67.9 (1%) | 0.02 (0%) | 0.00 (0%) |
| Candy, chocolate | 513 (6%) | 0.56 (6%) | 0.00 (0%) | 539 (7%) | 0.61 (8%) | 0.00 (0%) | 481 (5%) | 0.49 (5%) | 0.00 (0%) |
| Salty snacks | 210 (2%) | 0.29 (3%) | 0.00 (0%) | 183 (2%) | 0.10 (1%) | 0.00 (0%) | 242 (2%) | 0.13 (1%) | 0.00 (0%) |
| **FAT** | **203 (2%)** | **0.00 (0%)** | **0.00 (0%)** | **202 (3%)** | **0.00 (0%)** | **0.00 (0%)** | **205 (2%)** | **0.00 (0%)** | **0.00 (0%)** |
| Butter, margarine, veg oils | 203 (2%) | 0.00 (0%) | 0.00 (0%) | 202 (3%) | 0.00 (0%) | 0.00 (0%) | 205 (2%) | 0.00 (0%) | 0.00 (0%) |
| **OTHERS** | **329 (4%)** | **0.56 (6%)** | **0.00 (0%)** | **314 (4%)** | **0.17 (2%)** | **0.00 (0%)** | **347 (3%)** | **0.20 (2%)** | **0.00 (0%)** |
| Sauces, dressings etc. | 292 (3%) | 0.27 (3%) | 0.00 (0%) | 295 (4%) | 0.14 (2%) | 0.00 (0%) | 288 (3%) | 0.15 (2%) | 0.00 (0%) |
| Spices, broth, protein powder/bars | 37.3 (0%) | 0.29 (3%) | 0.00 (0%) | 19.6 (0%) | 0.03 (0%) | 0.00 (0%) | 59.2 (1%) | 0.05 (1%) | 0.00 (0%) |

**Table S4.** OR with 95% CI of iron deficiency by intakes of food groups among girls in the iron status subsample in Riksmaten Adolescents 2016–17.

|  |  | n low/not low | Adjusted model 1^a^ | | Adjusted model 2^b^ | |
| --- | --- | --- | --- | --- | --- | --- |
|  |  | (% low) | OR | 95% CI | OR | 95% CI |
| Red meat, g per day | |  |  |  |  |  |
|  | Q1 ≤ 57 | 43/98 (30) | 1.00 | reference | 1.00 | reference |
|  | Q2 57–75 | 39/104 (27) | 1.07 | 0.70; 1.62 | 1.04 | 0.66; 1.64 |
|  | Q3 75–92 | 26/117 (20) | 0.67 | 0.35; 1.29 | 0.61 | 0.33; 1.16 |
|  | Q4 >92 | 26/117 (20) | 0.64 | 0.40; 1.02 | 0.61 | 0.37; 0.99 |
| Cereal products, g per day | |  |  |  |  |  |
|  | Q1 ≤ 187 | 34/111 (23) | 1.00 | reference | 1.00 | reference |
|  | Q2 188–224 | 42/101 (29) | 1.26 | 0.63; 2.53 | 1.32 | 0.64; 2.72 |
|  | Q3 225–271 | 27/115 (23) | 0.81 | 0.43; 1.53 | 0.87 | 0.45; 1.68 |
|  | Q4 >271 | 31/109 (22) | 0.73 | 0.37; 1.44 | 0.84 | 0.42; 1.69 |
| Fruit and vegetables, g per day | |  |  |  |  |  |
|  | Q1 ≤ 169 | 30/113 (21) | 1.00 | reference | 1.00 | reference |
|  | Q2 170–229 | 34/107 (24) | 1.02 | 0.57; 1.81 | 1.09 | 0.62; 1.91 |
|  | Q3 230–306 | 36/108 (25) | 1.12 | 0.63; 2.01 | 1.20 | 0.67; 2.16 |
|  | Q4 > 306 | 34/108 (24) | 0.94 | 0.54; 1.62 | 1.05 | 0.57; 1.93 |
| Dairy products g per day | |  |  |  |  |  |
|  | Q1 ≤ 184 | 33/107 (24) | 1.00 | reference | 1.00 | reference |
|  | Q2 185–296 | 38/108 (26) | 1.32 | 0.73; 2.39 | 1.37 | 0.73; 2.57 |
|  | Q3 297–458 | 35/106 (25) | 1.29 | 0.65; 2.56 | 1.40 | 0.67; 2.91 |
|  | Q4 > 458 | 28/115 (20) | 1.12 | 0.61; 2.06 | 1.26 | 0.66; 2.41 |
| Sweet foods, g per day | |  |  |  |  |  |
|  | Q1 ≤ 66 | 28/113 (20) | 1.00 | reference | 1.00 | reference |
|  | Q2 67–86 | 33/109 (23) | 0.85 | 0.50; 1.47 | 0.83 | 0.48; 1.45 |
|  | Q3 87–106 | 34/110 (24) | 1.00 | 0.59; 1.69 | 1.02 | 0.57; 1.84 |
|  | Q4 > 106 | 39/104 (27) | 1.24 | 0.69; 2.24 | 1.25 | 0.69; 2.29 |
| Sugar-sweetened beverages, g per day | |  |  |  |  |  |
|  | Q1 ≤151 | 36/100 (26) | 1.00 | reference | 1.00 | reference |
|  | Q2 152–244 | 26/126 (17) | 0.64 | 0.37; 1.14 | 0.64 | 0.36; 1.14 |
|  | Q3 245–364 | 27/111 (20) | 0.81 | 0.46; 1.45 | 0.81 | 0.45; 1.46 |
|  | Q4 > 364 | 45/144 (31) | 1.19 | 0.67; 2.11 | 1.15 | 0.64; 2.06 |
| ^a^ Model adjusted for school grade, country of birth (Sweden/outside Sweden), menstruation (yes/no), and includes school as cluster variable. ^b^ Model additionally including mutual adjustment for the different food groups (as continuous intake in g/d). | | | | | | |

**Table S5.** OR with 95% CI of iron deficiency by intakes of food groups among boys in the iron status subsample in Riksmaten Adolescents 2016–17.

|  |  | n low/not low | Adjusted model 1^a^ | | Adjusted model 2^b^ | |
| --- | --- | --- | --- | --- | --- | --- |
|  |  | (% low) | OR | 95% CI | OR | 95% CI |
| Red meat, g per day | |  |  |  |  |  |
|  | Q1 ≤ 95 | 8/105 (7) | 1.00 | reference | 1.00 | reference |
|  | Q2 95–120 | 8/104 (7) | 0.85 | 0.20; 3.62 | 0.72 | 0.13; 3.86 |
|  | Q3 120–141 | 7/106 (6) | 0.74 | 0.26; 2.13 | 0.54 | 0.17; 1.77 |
|  | Q4 >141 | 4/109 (4) | 0.57 | 0.14; 2.21 | 0.35 | 0.08; 1.50 |
| Cereal products, g per day | |  |  |  |  |  |
|  | Q1 ≤ 226 | 6/106 (5) | 1.00 | reference | 1.00 | reference |
|  | Q2 227–281 | 7/106 (6) | 1.07 | 0.42; 2.75 | 1.13 | 0.39; 3.26 |
|  | Q3 282–353 | 5/109 (4) | 0.73 | 0.27; 2.01 | 0.70 | 0.23; 2.11 |
|  | Q4 >353 | 9/103 (8) | 1.18 | 0.46; 3.07 | 1.10 | 0.39; 3.14 |
| Fruit and vegetables, g per day | |  |  |  |  |  |
|  | Q1 ≤ 160 | 8/105 (7) | 1.00 | reference | 1.00 | reference |
|  | Q2 161–207 | 4/109 (4) | 0.53 | 0.14; 2.04 | 0.54 | 0.14; 2.13 |
|  | Q3 208–290 | 5/108 (4) | 0.73 | 0.21; 2.60 | 0.79 | 0.23; 2.67 |
|  | Q4 >290 | 10/102 (9) | 1.14 | 0.39; 3.28 | 1.09 | 0.34; 3.49 |
| Dairy products, g per day | |  |  |  |  |  |
|  | Q1 ≤ 285 | 4/108 (4) | 1.00 | reference | 1.00 | reference |
|  | Q2 286–468 | 6/108 (5) | 1.42 | 0.60; 3.38 | 1.47 | 0.56; 3.85 |
|  | Q3 469–710 | 3/109 (3) | 0.74 | 0.14; 3.89 | 0.85 | 0.16; 4.49 |
|  | Q4 >710 | 14/99 (12) | 3.27 | 1.31; 8.14 | 4.20 | 1.63; 10.83 |
| Sweet foods, g per day | |  |  |  |  |  |
|  | Q1 ≤ 46 | 3/109 (3) | 1.00 | reference | 1.00 | reference |
|  | Q2 47–79 | 8/106 (7) | 3.03 | 0.87; 10.60 | 3.71 | 0.87; 15.73 |
|  | Q3 80–112 | 8/104 (7) | 2.89 | 0.65; 12.89 | 3.05 | 0.52; 17.82 |
|  | Q4 >112 | 8/105 (7) | 2.31 | 0.54; 9.99 | 2.66 | 0.51; 13.80 |
| Sugar-sweetened beverages, g per day | |  |  |  |  |  |
|  | Q1 ≤195 | 4/106 (4) | 1.00 | reference | 1.00 | reference |
|  | Q2 196–341 | 7/108 (6) | 2..00 | 0.58; 6.94 | 2.63 | 0.68; 10.15 |
|  | Q3 342–504 | 11/102 (10) | 2.75 | 0.83; 9.18 | 4.01 | 0.97; 16.60 |
|  | Q4 >504 | 5/108 (4) | 1.31 | 0.54; 3.19 | 2.06 | 0.67; 6.34 |
| ^a^ Model adjusted for school grade, country of birth (Sweden/outside Sweden), and includes school as cluster variable. ^b^ Model additionally including mutual adjustment for the different food groups (as continuous intake in g/d). | | | | | | |

**Table S6.** Intake of red meat and other foods in the iron status subsample from Riksmaten Adolescents 2016-2017.

|  |  | **All** | | **Grade 5** | | **Grade 8** | | **Grade 11** | |
| --- | --- | --- | --- | --- | --- | --- | --- | --- | --- |
|  |  | **Girls n=579** | **Boys n=451** | **Girls n=164** | **Boys n=156** | **Girls n=222** | **Boys n=169** | **Girls n=193** | **Boys n=126** |
| Red meat^a^ | |  |  |  |  |  |  |  |  |
|  | g/d, mean (SD) | 73 (30) | 121 (41) | 81 (22) | 107 (38) | 70 (30) | 120 (28) | 71 (33) | 140 (51) |
|  | g/d, median (Q1, Q3) | 75 (57, 92) | 121 (95, 141) | 78 (68, 93) | 107 (83, 133) | 71 (52, 88) | 122 (106, 136) | 73 (52, 95) | 135 (106, 171) |
|  | g/10 MJ, mean (SD) | 92 (38) | 121 (41) | 107 (35) | 126 (42) | 86 (36) | 108 (27) | 87 (40) | 131 (49) |
|  | g/10 MJ, median (Q1, Q3) | 94 (72, 116) | 117 (95, 144) | 100 (87, 124) | 127 (95, 159) | 88 (64, 111) | 108 (93, 125) | 89 (63, 116) | 128 (99, 155) |
| Cereal products | |  |  |  |  |  |  |  |  |
|  | g/d, mean (SD) | 232 (69) | 299 (101) | 228 (62) | 260 (65) | 238 (79) | 321 (111) | 229 (62) | 316 (111) |
|  | g/d, median (Q1, Q3) | 225 (187, 271) | 281 (226, 353) | 225 (186, 261) | 257 (208, 297) | 226 (187, 282) | 307 (249, 371) | 223 (188, 265) | 300 (240, 377) |
|  | g/10 MJ, mean (SD) | 293 (85) | 297 (94) | 297 (80) | 311 (80) | 296 (89) | 286 (91) | 287 (84) | 295 (112) |
|  | g/10 MJ, median (Q1, Q3) | 281 (237, 345) | 286 (230, 352) | 298 (246, 343) | 305 (250, 364) | 277 (235, 355) | 274 (222, 336) | 274 (234, 334) | 280 (226, 334) |
| Fruit and vegetables | |  |  |  |  |  |  |  |  |
|  | g/d, mean (SD) | 247 (115) | 229 (109) | 211 (96) | 196 (79) | 258 (109) | 247 (128) | 264 (131) | 246 (104) |
|  | g/d, median (Q1, Q3) | 230 (170, 306) | 208 (161, 290) | 199 (139, 255) | 181 (146, 236) | 245 (179, 325) | 219 (172, 305) | 243 (179, 320) | 233 (174, 319) |
|  | g/10 MJ, mean (SD) | 309 (137) | 227 (99) | 272 (114) | 234 (96) | 322 (136) | 219 (100) | 325 (150) | 230 (102) |
|  | g/10 MJ, median (Q1, Q3) | 291 (213, 395) | 215 (158, 284) | 258 (192, 331) | 223 (166, 291) | 308 (221, 398) | 199 (156, 266) | 304 (222, 419) | 220 (149, 290) |
| Dairy products^b^ | |  |  |  |  |  |  |  |  |
|  | g/d, mean (SD) | 340 (211) | 530 (323) | 411 (222) | 497 (268) | 365 (226) | 642 (367) | 253 (143) | 419 (274) |
|  | g/d, median (Q1, Q3) | 297 (185, 458) | 469 (286, 710) | 379 (249, 541) | 467 (289, 647) | 314 (193, 517) | 577 (363, 875) | 227 (149, 345) | 371 (211, 589) |
|  | g/10 MJ, mean (SD) | 425 (253) | 520 (291) | 530 (272) | 580 (276) | 448 (257) | 564 (304) | 310 (175) | 387 (249) |
|  | g/10 MJ, median (Q1, Q3) | 382 (233, 569) | 494 (294, 706) | 509 (329, 690) | 567 (339, 773) | 407 (248, 623) | 529 (354, 723) | 277 (195, 416) | 305 (208, 557) |
| Sweet foods | |  |  |  |  |  |  |  |  |
|  | g/d, mean (SD) | 87 (29) | 86 (51) | 81 (30) | 73 (35) | 92 (27) | 102 (59) | 86 (30) | 82 (49) |
|  | g/d, median (Q1, Q3) | 87 (67, 106) | 80 (47, 112) | 80 (55, 101) | 71 (42, 95) | 91 (73, 108) | 92 (61, 135) | 86 (66, 106) | 72 (38, 111) |
|  | g/10 MJ, mean (SD) | 108 (32) | 84 (42) | 104 (34) | 87 (40) | 114 (29) | 90 (44) | 104 (33) | 74 (40) |
|  | g/10 MJ, median (Q1, Q3) | 109 (86, 129) | 84 (51, 110) | 105 (78, 131) | 86 (55, 109) | 114 (97, 133) | 89 (55, 121) | 102 (83, 123) | 67 (39, 101) |
| Sugar-sweetened beverages | |  |  |  |  |  |  |  |  |
|  | g/d, mean (SD) | 281 (173) | 379 (233) | 259 (87) | 274 (146) | 255 (156) | 378 (165) | 328 (227) | 509 (318) |
|  | g/d, median (Q1, Q3) | 245 (152, 364) | 342 (196, 504) | 243 (225, 348) | 250 (164, 364) | 234 (125, 362) | 383 (247, 512) | 282 (159, 433) | 436 (263, 669) |
|  | g/10 MJ, mean (SD) | 351 (203) | 371 (204) | 344 (130) | 330 (177) | 316 (185) | 341 (159) | 398 (258) | 463 (256) |
|  | g/10 MJ, median (Q1, Q3) | 320 (200, 465) | 340 (211, 473) | 329 (263, 431 | 313 (180, 450) | 287 (155, 431) | 327 (219, 450) | 361 (209, 533) | 418 (263, 624) |
| ^a^ Red meat refers to both unprocessed and processed red meat. ^b^ Dairy products including cheese (but not butter and ice-cream) | | | | | | | | | |
